# Synthesis of uniform single layer WS_2_ for tunable photoluminescence

**DOI:** 10.1038/s41598-017-16251-2

**Published:** 2017-11-23

**Authors:** Juhong Park, Min Su Kim, Eunho Cha, Jeongyong Kim, Wonbong Choi

**Affiliations:** 1 Department of Materials Science and Engineering, Department of Mechanical and Energy Engineering, University of North Texas, Denton, Texas 76207 United States; 2 Center for Integrated Nanostructure Physics (CINAP), Institute for Basic Science (IBS), Sungkyunkwan University, Suwon, 16419 Republic of Korea; 3 Department of Energy Science, Sungkyunkwan University (SKKU), Suwon, 16419 Republic of Korea

## Abstract

Two-dimensional transition metal dichalcogenides (2D TMDs) have gained great interest due to their unique tunable bandgap as a function of the number of layers. Especially, single-layer tungsten disulfides (WS_2_) is a direct band gap semiconductor with a gap of 2.1 eV featuring strong photoluminescence and large exciton binding energy. Although synthesis of MoS_2_ and their layer dependent properties have been studied rigorously, little attention has been paid to the formation of single-layer WS_2_ and its layer dependent properties. Here we report the scalable synthesis of uniform single-layer WS_2_ film by a two-step chemical vapor deposition (CVD) method followed by a laser thinning process. The PL intensity increases six-fold, while the PL peak shifts from 1.92 eV to 1.97 eV during the laser thinning from few-layers to single-layer. We find from the analysis of exciton complexes that both a neutral exciton and a trion increases with decreasing WS_2_ film thickness; however, the neutral exciton is predominant in single-layer WS_2_. The binding energies of trion and biexciton for single-layer WS_2_ are experimentally characterized at 35 meV and 60 meV, respectively. The tunable optical properties by precise control of WS_2_ layers could empower a great deal of flexibility in designing atomically thin optoelectronic devices.

## Introduction

Atomic layer two-dimensional transition metal dichalcogenides (2D TMDs) have garnered many interests for their unique electrical and optical properties including excellent electron mobility, high photoluminescence, semiconductor at atomic scale, and tunable band gap^1–3^. Recent studies have shown that single-layer TMDs exhibit direct band gap property that is accompanied by strong photoluminescence (PL) emission and large exciton binding energy; thus, they are promising materials for fundamental studies as well as next-generation ultra-thin opto-electronic devices^4,5^. Among the 2D TMDs, most researches have focused on MoS_2_ in hopes of finding new properties and potential applications; however, little attention has been paid to WS_2_. Single-layer WS_2_ has a direct band gap of 2.1 eV^6^ and a strong quantum yield of ~6% (single-layer MoS_2_ yields ~0.1%)^7^, whereas few-layer WS_2_ is an indirect band gap semiconductor with a bandgap of 1.35eV^8^. Few attempts have been made to synthesize large scale single-layer WS_2_ film. Song *et al*.^9^ presented fabrication of layer-controlled WS_2_ by sulfurization of WO_3_ film using atomic layer deposition (ALD), and Yun *et al*.^10^ reported centimeter-scale single-layer WS_2_ on gold foil by using chemical vapor deposition (CVD). However, their PL spectra within the single-layer WS_2_ film were spatially nonuniform. In our previous report^11^, we demonstrated centimeter scale WS_2_ film by the two-step process of tungsten deposition followed by sulfurization in a low-pressure CVD. Despite the successful synthesis of the large scale WS_2_ film, the film exhibits both single- and few-layer of WS_2_; also, the single-layer WS_2_ does not show uniformity. Thus, several post-treatment approaches were attempted to control the number of layers for any 2D TMDs. For example, Castellanos-Gomez *et al*.^12^ reported the thinning of expoliated few-layer MoS_2_ film down to a single-layer by using a focused laser beam. Venkatakrishnan *et al*.^13^ reported a laser thinning of WS_2_ flake down to single-layer with revealing improvement of the fluorescence emission intensity and micro-encryption by surface modification. Ni *et al*.^14^ used a plasma technique for layer by layer etching of mechanically exfoliated MoS_2_ film; however, this technique requires physical mask for selective etching on TMDs film. No systematic study for fabrication of uniform single-layer WS_2_ and its related opto-electronic properties have been reported.

One of the peculiar features of single-layer TMDs is strong excitonic effect caused by their absence of interlayer coupling and the lack of inversion symmetry, which mostly account for the interesting PL emission property^15,16^. The excitons are generated by electron-hole pairs in a single-layer TMDs that has the large binding energies ranging from 0.3 to 1.0 eV, which is attributed to their strong Coulomb attraction between charged particles^17,18^. In particular, photoexcitation in 2D TMDs leads to the formation of multi-carrier bound states because excitons can interact with free electrons^19,20^. Such interaction forms the exciton complexes including trions, a localized excitons consists of three charged quasiparticles (*e.g*., a negative trion consists of two electrons and one hole and a positive trion consists of two holes and one electron). The interaction of charged carriers and excitons controls the optical properties of TMDs^21^. Hence, the first step is to understand the behavior of exciton complexes in WS_2_ for practical applications in opto-electronic devices as well as for the fundamental physics of emergent new materials. Furthermore, the exact values for the binding energy of the excitons in WS_2_ are still debated and the behavior of exciton complexes with respect to the WS_2_ layers remains unexplored.

In this regard, we have employed high-power laser processing to fabricate a uniform single-layer WS_2_ from the few-layer WS_2_ synthesized by a scalable two-step CVD method. Atomically uniform single-layer WS_2_ was successfully synthesized by the two-step CVD method followed by a laser thinning method. The behavior of exciton complexes with the number of WS_2_ layer was investigated during the laser thinning process. We found that the PL intensity has increased linearly (up to 6 times higher) as the number of WS_2_ layer decreased. In particular, the dominant exciton in single-layer WS_2_ is neutral exciton, while trion is dominant in few-layer WS_2_ as analyzed by PL spectrum. These changes of exciton intensity contribute largely to the PL spectra of WS_2_ film; such phenomenon is invaluable to engineer the opto-electronic properties of 2D WS_2_.

## Results

### Synthesis and characterization of few-layer WS_2_

**Figure 1:**
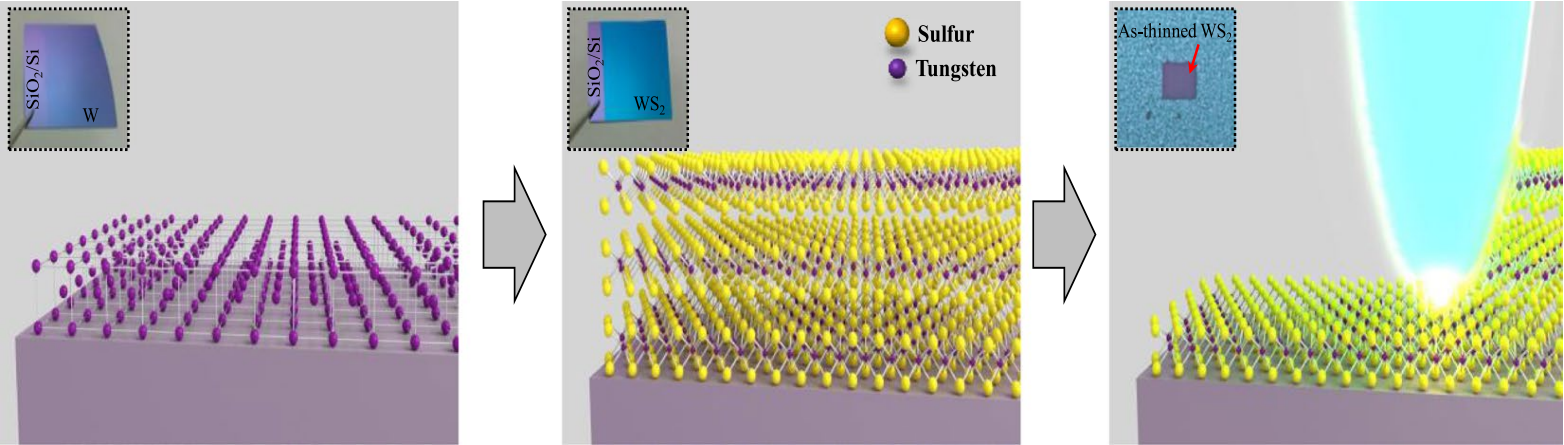
Schematics illustrating the two-step method for few-layer WS_2_ film growth and the laser thinning process for single-layer WS_2_ fabrication (Insets show the optical images of as-deposited W film, WS_2_ film after sulfurization of W film, and laser thinned WS_2_ film on SiO_2_/Si substrate).

The synthesis of uniform few atomic layer 2D TMDs was presented in our previous report by the two-step method^1,22^. Here we introduce a large-scale single-layer WS_2_ film synthesis by using the two-step method followed by a laser thinning process. Schematic of the overall process and optical images of the sample in each step are presented in Fig. 1.

**Figure 2:**
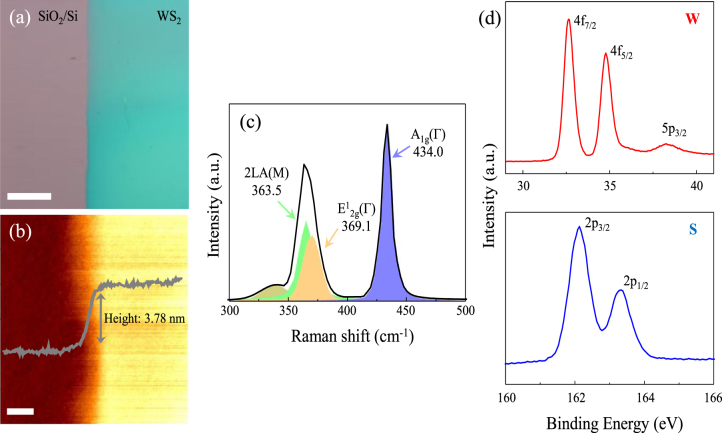
(**a**) Optical image of the few-layer WS_2_ film on SiO_2_/Si substrate. Scale bar: 100 μm. (**b**) Height profile and image of the WS_2_ film (Scale bar: 5 μm). (**c**) Raman spectrum of the WS_2_ film using the 514 nm laser excitation and its Lorentzian peak fits. (**d**) XPS data of the W 4 f and S 2p core levels of the WS_2_ film.

The synthesized WS_2_ film was characterized by AFM, Raman spectroscopy, PL, and XPS. The optical image of as-synthesized few-layer WS_2_ film on a SiO_2_/Si substrate indicates uniform and large-scale growth of WS_2_ film (Fig. 2a). The thickness of WS_2_ film is estimated to be ~3.78 nm (Fig. 2b), which is 4–5 layers of WS_2_, as confirmed by the AFM in the previous studies^6,23^. Figure 2c presents the Raman spectrum of as-synthesized few-layer WS_2_ film (measured at 514 nm excitation laser line). The Raman spectrum is governed by the first-order modes: E^1^_2g_ (Г) at 369.1 cm^−1^ and A_1g_ (Г) at 434.0 cm^−1^; however, the intensity of the second order mode of 2LA (M) at 363.5 cm^−1^ is also very high for WS_2_. Even though the 2LA (M) mode is overlapped with the E^1^_2g_ mode, the peak is separated their individual contributions by the Lorentzian fitting. The frequency difference (△k) between 2LA (M) and A_1g_ as well as E^1^_2g_ and A_1g_ modes are 70.5 cm^−1^ and 64.9 cm^−1^, respectively. Thus, it is confirmed that the as-synthesized film is 4–5 layers of WS_2_^6,24^. We also characterized the WS_2_ film using XPS (Fig. 2d). The 4 f core-level spectrum represents three peaks at 32.6, 34.8, 38.2 eV corresponding to the W 4f_7/2_, W 4f_5/2_, and W 5p_3/2_ state, respectively. The S 2p core-level shows two peaks at 162.1 and 163.3 eV, which match with the S 2p_3/2_ and S 2p_1/2_ states, respectively. Based on the XPS data, an excellent stoichiometry of the atomic WS_2_ film is realized by the calculated S (66.7%) to W (33.3%) ratio of 2^25^.

### Characterization of single-layer WS_2_ fabricated by laser thinning method

**Figure 3:**
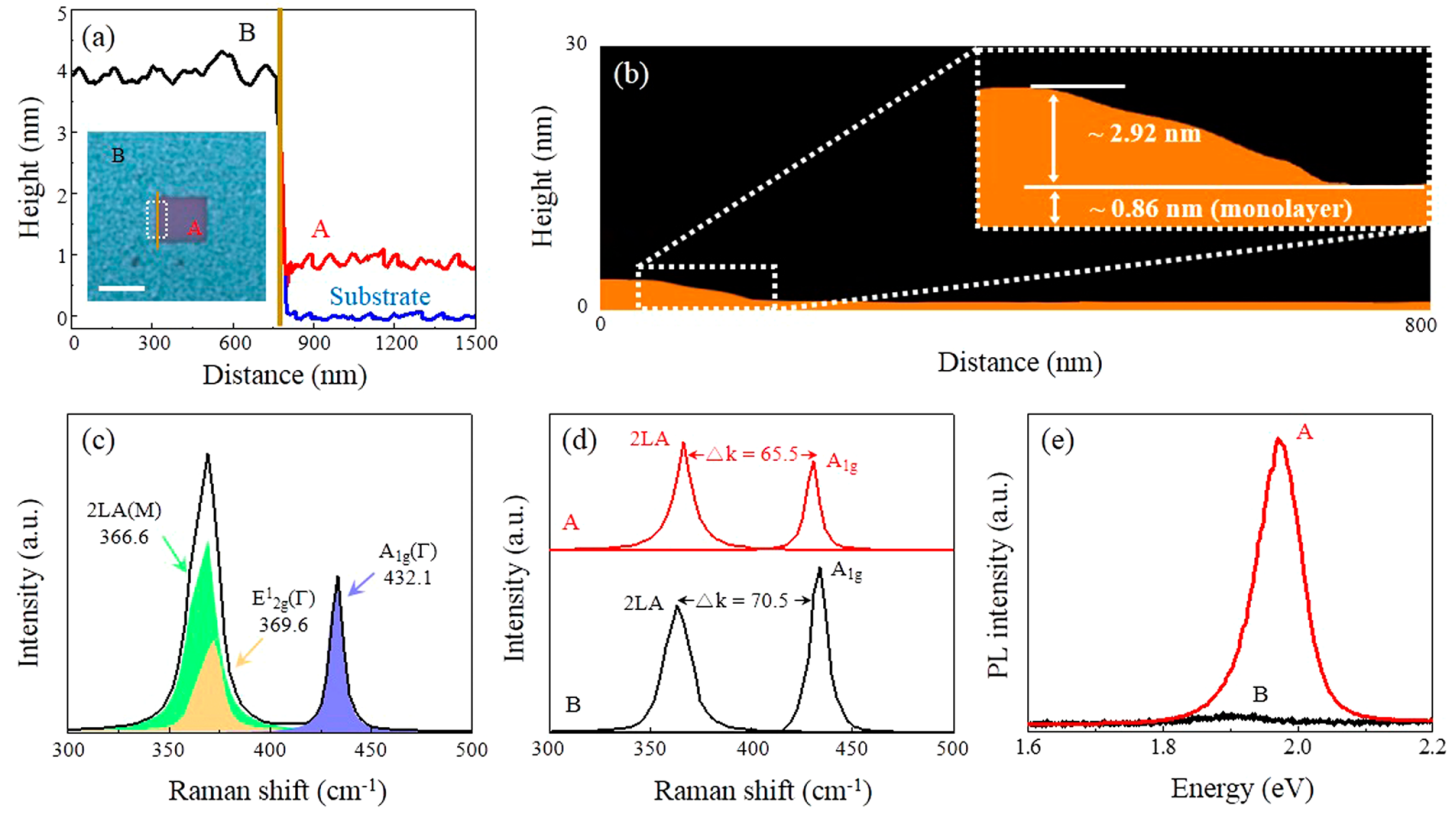
(**a**) AFM height profile of laser irradiated region (A), non-irradiated region (B), and substrate (ground): the measured thickness between A and B is ~2.88 nm; between the substrate and A is ~0.9 nm (Inset shows the optical image of the laser irradiated area for 15 seconds on 5 × 5 μm, scale bar: 5 μm). (**b**) An average AFM height profile of the laser irradiated region (A) shows the etched thickness of ~2.92 nm. (**c**) Raman spectrum of a single-layer WS_2_ film (A) using the 514 nm laser excitation and its Lorentzian peak fits. (**d**) Raman spectra showing the peak distance (△k) between 2LA and A_1g_ measuring at 65.5 cm^−1^ and 70.5 cm^−1^ for regions A and B, respectively. (**e**) PL spectra of region A and B showing ~6 times increase of PL emission intensity and ~0.05 eV shift of PL peak position by the laser irradiation.

The as-synthesized WS_2_ film was irradiated by a scanning laser to thin the few-layer WS_2_ down to a single-layer. Inset of Fig. 3a shows the optical image of WS_2_ film after laser irradiation for 10 seconds; the image represents the laser covering 5 μm × 5 μm area. Color contrast is observed for the laser irradiated region (A) showing uniform and reduced thickness. The relative thickness difference after the laser irradiation (Fig. 3a) is ~2.88 nm; thus, the thickness of laser irradiation region is ~0.9 nm which corresponds to a single-layer WS_2_. The detailed surface profile of laser irradiated region is investigated by AFM (Fig. 3b). Remarkably, the laser beam can etch up to ~2.92 nm uniformly over the as-synthesized ~3.78 nm thick WS_2_ film. It should be noted that the edge of the sidewalls is not flat. This is attributed to the Gaussian intensity profile of the confocal laser. The sidewall could be removed by overlapping the irradiated laser spots. After laser irradiation, Raman and PL measurements were performed with a reduced laser power of 200 μW to the irradiated area of 5 μm × 5 μm (Fig. 3c). The Raman spectra show the 2LA (M), E^1^_2g_ (Г), and A_1g_ (Г) modes at 366.6 cm^−1^, 369.6 cm^−1^, and 432.1 cm^−1^, respectively.

**Figure 4:**
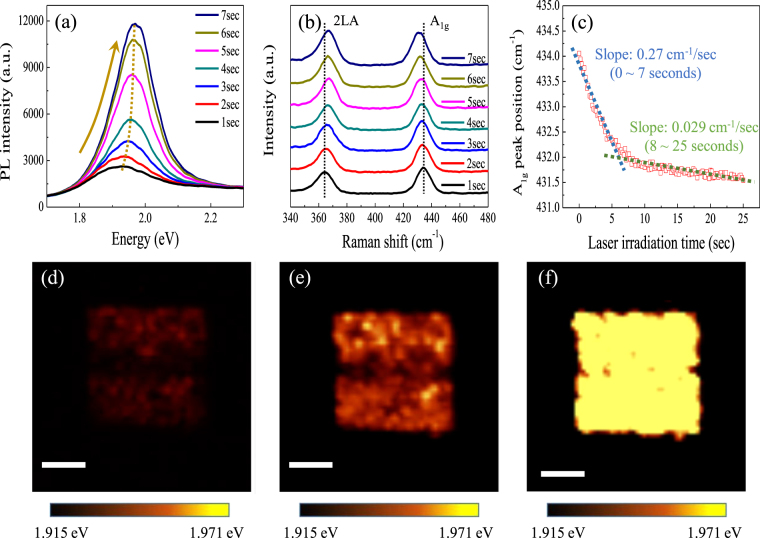
(**a**) PL and (**b**) Raman spectra as a function of the laser irradiation time from 1 to 7 seconds. (**c**) Raman frequencies recorded at various laser irradiation time (from 1 to 25 seconds) for A_1g_ mode. PL peak position map of the laser irradiated area of 5 μm × 5 μm after (**d**) 2 seconds, (**e**) 5 seconds, and (**f**) 10 seconds of laser irradiation. Scale bar: 2 μm.

Figure 3d presents the 2LA (M) and A_1g_ modes of both laser irradiated (A) and non-irradiated (B) regions on WS_2_ film. The frequency differences (△k) between two modes changed from 70.5 cm^−1^ to 65.5 cm^−1^; also, the relative intensity of *I*_2LA_/*I*_A1g_ increased from 0.77 to 1.1. Berkdemir *et al*.^6^ reported that frequency differences (△k) and the intensity ratios investigated by Raman spectra (λ_exc_ = 514 nm) for thin WS_2_ film are modifiable by the number of layers. Particularly, single-layer WS_2_ shows △k of 65.2 cm^−1^ and *I*_2LA_/*I*_A1g_ of 2.1; those values are slightly different from the results of our single-layer WS_2_ fabricated by laser irradiation. The differences are attributed to the unetched few-layer WS_2_ as illustrated in the height profile (Fig. 3b). Figure 3e indicates PL intensities for both regions A (laser-irradiated) and B (non-irradiated) in Fig. 3a. After laser irradiation, PL intensity increases substantially up to 6 times; in addition, the PL peak position is shifted from 1.92 eV to 1.97 eV. The 1.97 eV PL peak position corresponds to single-layer WS_2_^26,27^. Based on this approach, wafer scale laser-thinned single-layer WS_2_ film could be readily fabricated by using a scanning laser beam irradiation. We employed *in situ* confocal PL and Raman spectroscopy to investigate the variation in the PL and Raman spectra with respect to the laser irradiation time. Figure 4a depicts the PL spectra of the WS_2_ film measured with laser irradiation times (from 0 to 7 seconds) under ambient conditions. The PL intensity increases steadily as a function of the laser irradiation time of up to 7 seconds in which the PL intensity reaches maximum; as a result, the PL peak position is shifted from 1.92 eV to 1.97 eV, (dotted line in Fig. 4a). Thus, the precise thinning of the as-synthesized few-layer WS_2_ film by the laser irradiation is evidenced by the increase in PL peak intensity and the shift in position. Figure 4b indicates Raman spectra of WS_2_ film recorded at different laser irradiation time. The relative intensity (*I*_2LA_/*I*_A1g_) is gradually changed from 0.8 for 1 second to 1.1 for 7 seconds; also, the frequency difference between 2LA (M) and A_1g_ (Г) modes is reduced to 65.5 cm^−1^ for 7 seconds of irradiation time. The constant change of PL and Raman spectra with laser irradiation time reveals that the few-layer WS_2_ film is continuously thinned with the laser time. The Raman peak position shift in A_1g_ mode shows an interesting phenomenon with regard to self-limited etching behavior. Figure 4c shows the A_1g_ mode shift as a function of laser irradiation time (from 0 to 25 seconds). It is noted that there exist two regimes: the region exposed for 0–7 seconds shows larger slope than the region exposed for 8–25 seconds. As a result, it is expected that the top WS_2_ layers are etched entirely by the laser irradiation of 7 seconds, whereas the bottom single-layer WS_2_ remains unetched even after 7 seconds laser irradiation. The lower slope of the A_1g_ mode after 7 seconds is attributed to the local strains or disorders caused by the continuous laser irradiation^28,29^. PL and Raman spectra (Fig. 4a and b) verify that the single-layer WS_2_ film is already achieved after laser irradiation for 7 seconds; thus, the etching rate of WS_2_ film is ~0.42 nm/sec calculated by etched thickness (2.92 nm in Fig. 3b) and laser irradiation time (7 seconds).

To demonstrate the fabrication of single-layer WS_2_ film with high uniformity, PL mapping with a PL peak position is carried out on the laser irradiated area (Fig. 4d–f). It is observed that the PL peak variation is uneven for 2 and 5 second irradiation time. However, the spatial uniformity increases significantly after laser irradiation for 10 seconds as shown in Fig. 4f. This uniform PL peak position demonstrates that the formation of uniform single-layer WS_2_ film was achieved by the laser irradiation; also, further undesirable damage after forming the single-layer WS_2_ film was prevented^30^. Once the normally incident laser is absorbed on WS_2_ layers, the WS_2_ layers produce local heating on the plane. Gastellanos-Gomez *et al*.^12^ reported that the generated thermal energy mostly dissipate through the planer direction to the TMDs layers rather than the perpendicular direction which is bonded by weak van der Waals forces. Similarly, as reported by Han *et al*.^30^, the generated heat by light absorption is mainly accumulated on the upper graphene layers when a laser is induced on the film, while the SiO_2_/Si substrate plays an important role as a heat reservoir for the single-layer graphene to remain unetched. It is also reported that the heat conduction across 2D crystals-substrate interface is not negligible^31^. Initially, the heat propagates mostly along the basal plane of WS_2_ film due to higher thermal conductivity of the basal plane (124 W/mK) than the c-axis of WS_2_ (1.7 W/mK)^32^. When the thickness is getting reduced by the laser irradiation, the heat conduction across WS_2_-SiO_2_/Si substrate becomes dominant, and the SiO_2_/Si substrate plays a role as a heat sink. Therefore, the flat and uniform single-layer WS_2_ film could be produced by laser irradiation.

### Investigation on behavior of exciton complexes

**Figure 5:**
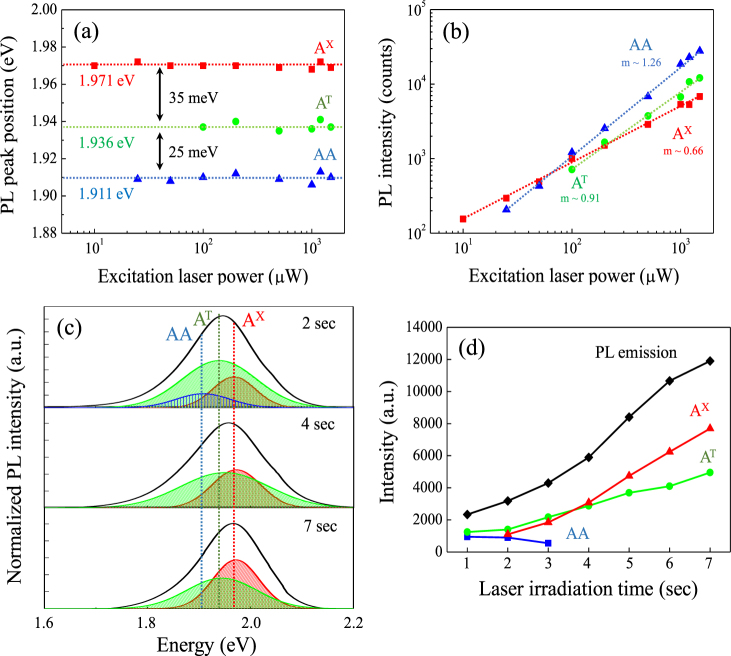
Laser power dependent PL (**a**) peak positions and (**b**) intensities of exciton complexes from the laser irradiated single-layer WS_2_. The *m* values indicate logarithmic values of each slope, and the dashed lines in (**a**) and (**b**) are guides for the eye to visualize. (c) Laser irradiation time dependent photoluminescence spectra and the deconvoluted excitation complexes for 2, 4, and 7 seconds laser irradiation and (**d**) the overall peak position of exciton complexes with laser irradiation time for1–7 seconds.

The change of PL spectra was reported to be associated with a transition from indirect band gap (few-layer WS_2_) to direct band gap (single-layer WS_2_). Because the origins of variation in relative contributions of exciton complexes (*i.e*. neutral exciton (A^X^), trion (A^T^), and biexciton (AA)) to PL emission have not been studied, we investigate the behavior of the exciton complexes depending on the numbers of WS_2_ layer. First, we analyze the PL intensities of exciton complexes as a function of laser power to determine the transition and binding energies of the exciton complexes (Fig. 5a and b) as per the definition of the energies^21^. These plots reveal that the estimated transition energies for A^X^, A^T^, and AA are 1.971, 1.936, and 1.911, respectively; also, the binding energies for A^T^, and AA are 35 meV and 60 meV, respectively. Those binding energies are consistent with the computationally simulated results of WS_2_^33–35^. Each excitons indicates different values of slope (*m*) regarding the increase of PL intensity with respect to laser power (Fig. 5b). Based on previous studies, the exponent of *m* = 1.2–1.9 is a typical value for AA^36,37^; in other words, AA has super-linear slopes because of the kinetics of excitation recombination and formation^38^. It is also important to note that there is an indication for the exponent of the A^X^ (*m* ~0.66) is half the value of the AA (*m* ~1.26)^39^. Therefore, the sub-linear values of *m* ~0.66 and *m* ~0.91 for A^X^ and A^T^, respectively, and the super-linear value of *m* ~1.26 for AA can verify the types of exciton for WS_2_^40^. Figure 5c and d indicates the PL spectra obtained from representative irradiation times of 1–7 seconds by decomposing them into A^X^, A^T^, and AA. For the increased laser irradiation of up to 7 seconds, the predominant exciton of the PL spectrum is changed from A^T^ to A^X^ (Fig. 5c). It is noted that the steady intensity of AA disappeared at 3 seconds of irradiation time; thus, only A^T^ and A^X^ affects the PL spectra of laser thinned WS_2_ film, whereas both A^T^ and A^X^ increase as the number of WS_2_ layers decrease by the longer laser irradiation time. The increased rate in the intensity of A^X^ is higher than A^T^ (Fig. 5d); therefore, the PL spectrum is shifted up to 1.97 eV for the single-layer.

We expect that the intensity variation for both A^T^ and A^X^ is affected by the addition of charge carriers (either by an electron or hole) absorbed by oxygen molecule during laser irradiation; also, it is assumed that the variation of charge carriers can directly modify the intensity of both A^T^ and A^X^ ^41,42^. Oh *et al*.^43^ reported the intensity of exciton complexes for single-layer MoS_2_ with various laser irradiation time under ambient conditions; here, the intensity of A^X^ increases dramatically in the first few minutes of laser irradiation due to the charge transfer of MoS_2_ to the adsorbed oxygen group induced by laser irradiation. Another report also presented a single-layer MoS_2_ treated by oxygen plasma that shows much improved PL spectrum because of the charge transfer from MoS_2_ to oxygen molecule on the sulfur vacancy^44^.

**Figure 6:**
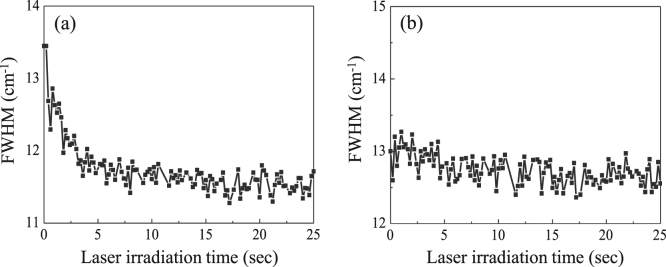
The full width at half-maximum (FWHM) of (**a**) A_1g_ mode and (**b**) E^1^_2g_ mode of WS_2_ as a function of the laser irradiation time (1–25 seconds).

To confirm the charge transfer effect, full width at half-maximum (FWHM) of the A_1g_ mode is measured with laser irradiation time (0–25 seconds) (Fig. 6a). The reduced FWHM of the A_1g_ mode during the WS_2_ film thinning is the evidence of p-doping (electron is moved from WS_2_ to oxygen molecule)^45^; otherwise, FWHM of the E^1^_2g_ mode is not widely variable as a function of laser irradiation time (Fig. 6b). Therefore, the charge transfer from WS_2_ to oxygen molecule induced by laser irradiation contributes to the increased ratio of A^X^ to A^T^ with the thinning of WS_2_. Interestingly, we observed the blue-shift of ~50 meV for the exciton peak as the thickness is reduced from few- to single-layer (Fig. 5c). Based on the previous experimental and theoretical studies, the quasiparticle band gap in 2D WS_2_ is expected to increase with thinning of 2D WS_2_; also, the binding energy of exciton complexes is predicted to increase with the thinning of WS_2_ film due to the enhanced electron-hole interaction by weak dielectric screening^46–50^. The increase in quasiparticle band gap and the exciton binding energy affects the exciton peak position; thus, the shift of exciton complexes is negligible with the decreasing number of WS_2_ layer. The blue-shifting of the exciton peak (measured ~50 meV) during the laser thinning process is consistent with the previous report^15^.

## Discussion

Precise control of WS_2_ layers is achieved by using two-step CVD synthesis method followed by a laser-thinning. The scalable synthesis of uniform single-layer WS_2_ film is confirmed by AFM thickness measurement (~0.9 nm), Raman frequencies difference (65.5 cm^−1^), and PL peak position (1.97 eV). Based on the variation of PL and Raman spectra with respect to laser irradiation time, the optimum laser irradiation time for the synthesis of a single-layer WS_2_ film is found to be 7 seconds. It is also realized that the single-layer WS_2_ is retained with additional laser irradiation time, which is attributed to the thermal energy dissipated through the substrate. The intensity of both neutral exciton and trion increases with the reduction of WS_2_ thickness; however, the biexciton appears to have no noticeable change. In particular, dominant exciton component in PL spectrum for the few-layer and single-layer WS_2_ is trion and neutral exciton, respectively. The observed binding energies of the exciton complexes for single-layer WS_2_ are 35 meV (trion) and 60 meV (biexciton). The tunable optical properties by precise control of WS_2_ layers and the understanding of their exciton complexes would lead to the design of novel optoelectronic devices.

## Methods

### Preparation of wafer scale thin WS_2_ film

Large-scale few-layer WS_2_ film was synthesized on a p-type silicon substrate (Boron doped; 0.001–0.005 Ω·cm) with 300 nm thick SiO_2_ by using two-step method involving magnetron sputtering for deposition of tungsten (W) film and chemical vapor deposition (CVD) for sulfurization of W film. For the first step, 99.99% purity W target (Plasmaterials) was used to deposit thin film of W. Sputtering W film was carried out for 10 seconds at room temperature. Subsequently, the W film was sulfurized in the CVD for 1 hour at 600 °C to transform the film into WS_2_. During sulfurization, the sulfur powder was heated separately at ~250 °C, and argon was used as a carrier gas to convey sulfur (S) vapor species toward the W films.

### Characterization of the WS_2_ film

Thickness and surface analysis of as-synthesized WS_2_ film were performed by atomic force microscopy (AFM) (Parks system, NX-10 model). X-ray photoelectron spectroscopy (XPS) (Thermo Scientific, ESCALAB250 model) was used for the chemical binding energies of W and S orbitals. A lab-made spectrometer combined with 514 nm wavelength and 200 μW power of a solid-state confocal laser microscope was used for PL and Raman spectroscopy measurements^40,51^. A 0.9 NA objective was used to focus the laser light which the lateral resolution was set at approximately 300 nm. Scattered light was gathered by the 0.9 NA objective and directed to a 50 cm long monochromater equipped with a cooled CCD.

### Condition of laser thinning process

A scanning laser from a lab-made spectroscope (laser wavelength of 514 nm with 2.5 mW power and 300 nm lateral resolution) was used to thin few-layer WS_2_ film down to single-layer by moving the laser over the WS_2_ film laterally by 300 nm for the next exposure. The total number of exposures is 256 times for 5 × 5 μm area, and the exposure time is 15 seconds for each laser irradiation. For a detailed study of exciton complexes variation depending on layer numbers, we used the same laser with increased laser exposure time from 0 to 25.6 seconds and measured the individual PL spectrum per each 0.2 second (total 128 measurements) under ambient conditions.
